# Enterovirus and Encephalitis

**DOI:** 10.3389/fmicb.2020.00261

**Published:** 2020-02-20

**Authors:** Bo-Shiun Chen, Hou-Chen Lee, Kuo-Ming Lee, Yu-Nong Gong, Shin-Ru Shih

**Affiliations:** ^1^Research Center for Emerging Viral Infections, College of Medicine, Chang Gung University, Taoyuan, Taiwan; ^2^Department of Neuroscience and Regenerative Medicine, Medical College of Georgia, Augusta University, Augusta, GA, United States; ^3^Department of Laboratory Medicine, Linkou Chang Gung Memorial Hospital, Taoyuan, Taiwan; ^4^Department of Medical Biotechnology and Laboratory Science, College of Medicine, Chang Gung University, Taoyuan, Taiwan; ^5^Research Center for Chinese Herbal Medicine, College of Human Ecology, Chang Gung University of Science and Technology, Taoyuan, Taiwan; ^6^Research Center for Food and Cosmetic Safety, College of Human Ecology, Chang Gung University of Science and Technology, Taoyuan, Taiwan; ^7^Graduate Institute of Health Industry Technology, College of Human Ecology, Chang Gung University of Science and Technology, Taoyuan, Taiwan

**Keywords:** enterovirus, encephalitis, CNS, Picornaviridae, RNA virus

## Abstract

Enterovirus-induced infection of the central nervous system (CNS) results in acute inflammation of the brain (encephalitis) and constitutes a significant global burden to human health. These viruses are thought to be highly cytolytic, therefore normal brain function could be greatly compromised following enteroviral infection of the CNS. A further layer of complexity is added by evidence showing that some enteroviruses may establish a persistent infection within the CNS and eventually lead to pathogenesis of certain neurodegenerative disorders. Interestingly, enterovirus encephalitis is particularly common among young children, suggesting a potential causal link between the development of the neuroimmune system and enteroviral neuroinvasion. Although the CNS involvement in enterovirus infections is a relatively rare complication, it represents a serious underlying cause of mortality. Here we review a selection of enteroviruses that infect the CNS and discuss recent advances in the characterization of these enteroviruses with regard to their routes of CNS infection, tropism, virulence, and immune responses.

## Introduction

Enteroviruses belong to the family Picornaviridae, a highly diverse group of small, non-enveloped, icosahedral-shaped viruses with single positive-strand RNA genomes. Based on the sequence diversity, they have been divided into 15 species: enterovirus A to L and rhinovirus A to C. Human enteroviruses containing four enterovirus species (A to D) and three rhinovirus species (A to C) infect millions of people worldwide every year. Although infections are frequently asymptomatic, human enteroviruses can cause a variety of symptoms comprising fever, headache, respiratory illness, sore throat, and, occasionally, vomiting and diarrhea. Importantly, several members of human enteroviruses are neurotropic pathogens with a wide range of clinical disorders ranging from aseptic meningitis to more severe encephalitis. In the United States, enterovirus has been shown to be the most common etiology of meningitis/encephalitis (Hasbun et al., 2017; Balada-Llasat et al., 2019). About 58% of the infected infants and children and 52% of the infected adults diagnosed with meningitis/encephalitis is due to enterovirus. In addition, age is highly associated with the clinical presentation with severe infections including CNS disease, myocarditis and sepsis-like illness occurring most frequently in neonates and infants. The best-known neurotropic enterovirus is the poliovirus (PV), which belongs to the species Enterovirus C and is believed to have almost been eradicated by vaccinations from circulation in human populations. Non-polio enteroviruses are also known to infect the CNS and account for the majority of recent enteroviral infections with neurological disorders. Among the non-polio enteroviruses, the species Enterovirus A like enterovirus 71 (EV-A71), coxsackievirus A6 (CV-A6), and CV-A16 (Goto et al., 2009; Xu et al., 2012; Huang Y. et al., 2015; Holmes et al., 2016; B’Krong et al., 2018; Suresh et al., 2018), the species Enterovirus B like CV-B1 (Sun et al., 2019), CV-B3 (Fan and Liu, 2019), CV-B5 (Mao et al., 2018), CV-A9, Echovirus 6 (E-6), E-7, E-11, and E-13 (Holmes et al., 2016; Chen et al., 2017; B’Krong et al., 2018; Suresh et al., 2018; Chen et al., 2019; Ramalho et al., 2019; Sun et al., 2019), the species Enterovirus C like CV-A24 (Tapparel et al., 2013; B’Krong et al., 2018; Suresh et al., 2018), and the species Enterovirus D like EV-D68 (Tapparel et al., 2013; Messacar et al., 2018) have been shown to involve the CNS and cause diverse neurological complications such as encephalitis, meningitis and acute flaccid paralysis (AFP) (Tapparel et al., 2013) (see Table 1 for details).

**TABLE 1:** Neurological symptoms of non-polio enteroviruses that involve the CNS.

Species	Serotype	CNS symptoms	References
A	EV-A71	E/ M/AFP	Huang Y. et al., 2015; Mao et al., 2016; B’Krong et al., 2018; Suresh et al., 2018; Ramalho et al., 2019
	EV-A76	AFP	Suresh et al., 2018
	EV-A90	AFP	Suresh et al., 2018
	CV-A2	M/AFP	Holmes et al., 2016; Suresh et al., 2018
	CV-A3	AFP	Suresh et al., 2018
	CV-A4	AFP	Suresh et al., 2018
	CV-A5	AFP	Suresh et al., 2018
	CV-A6	E/M/AFP	Huang Y. et al., 2015; Holmes et al., 2016; Suresh et al., 2018
	CV-A7	AFP	Suresh et al., 2018
	CV-A10	E/M/AFP	Huang Y. et al., 2015; Mao et al., 2016; B’Krong et al., 2018; Suresh et al., 2018
	CV-A12	AFP	B’Krong et al., 2018; Suresh et al., 2018
	CV-A14	AFP	Suresh et al., 2018
	CV-A16	E/M/AFP	Goto et al., 2009; Xu et al., 2012; Huang Y. et al., 2015; Holmes et al., 2016; Suresh et al., 2018
B	CV-A9	E/M/AFP	Holmes et al., 2016; Chen et al., 2017; B’Krong et al., 2018; Suresh et al., 2018; Ramalho et al., 2019; Sun et al., 2019
	CV-B1	E/M/AFP	Holmes et al., 2016; B’Krong et al., 2018; Suresh et al., 2018; Fan and Liu, 2019; Ramalho et al., 2019; Sun et al., 2019
	CV-B2	E/M/AFP	Holmes et al., 2016; B’Krong et al., 2018; Suresh et al., 2018; Fan and Liu, 2019; Ramalho et al., 2019
	CV-B3	E/M/AFP	Holmes et al., 2016; Mao et al., 2016; Mao et al., 2018; Suresh et al., 2018; Fan and Liu, 2019; Ramalho et al., 2019; Sun et al., 2019
	CV-B4	E/M/AFP	Holmes et al., 2016; Mao et al., 2016; B’Krong et al., 2018; Mao et al., 2018; Suresh et al., 2018; Sun et al., 2019
	CV-B5	E/M/AFP	Holmes et al., 2016; Mao et al., 2016; Chen et al., 2017; B’Krong et al., 2018; Mao et al., 2018; Suresh et al., 2018; Ramalho et al., 2019; Sun et al., 2019
	CV-B6	M/AFP	Tapparel et al., 2013; Suresh et al., 2018
	E-1	M/AFP	Tapparel et al., 2013; Suresh et al., 2018
	E-2	M/AFP	Tapparel et al., 2013; Suresh et al., 2018
	E-3	E/M/AFP	Tapparel et al., 2013; Suresh et al., 2018; Ramalho et al., 2019
	E-4	E/M/AFP	Tapparel et al., 2013; B’Krong et al., 2018; Suresh et al., 2018
	E-5	E/M/AFP	Tapparel et al., 2013; Holmes et al., 2016; Suresh et al., 2018
	E-6	E/M/AFP	Holmes et al., 2016; Chen et al., 2017; B’Krong et al., 2018; Suresh et al., 2018; Ramalho et al., 2019; Sun et al., 2019
	E-7	E/M/AFP	Tapparel et al., 2013; Holmes et al., 2016; Suresh et al., 2018; Ramalho et al., 2019
	E-9	E/M/AFP	Holmes et al., 2016; B’Krong et al., 2018; Suresh et al., 2018; Ramalho et al., 2019; Sun et al., 2019
	E-11	E/M/AFP	Holmes et al., 2016; Suresh et al., 2018; Ramalho et al., 2019; Sun et al., 2019
	E-12	M/AFP	Tapparel et al., 2013; B’Krong et al., 2018; Suresh et al., 2018
	E-13	E/M/AFP	Tapparel et al., 2013; Holmes et al., 2016; Suresh et al., 2018; Ramalho et al., 2019
	E-14	E/M/AFP	Chen et al., 2017; Suresh et al., 2018; Ramalho et al., 2019; Sun et al., 2019
	E-15	M/AFP	Suresh et al., 2018; Ramalho et al., 2019
	E-16	E/M/AFP	Holmes et al., 2016; B’Krong et al., 2018; Suresh et al., 2018; Sun et al., 2019
	E-17	E/M/AFP	Tapparel et al., 2013; Holmes et al., 2016; Suresh et al., 2018
	E-18	E/M/AFP	Holmes et al., 2016; Chen et al., 2017; B’Krong et al., 2018; Suresh et al., 2018; Chen et al., 2019; Ramalho et al., 2019; Sun et al., 2019
	E-19	E/M/AFP	Tapparel et al., 2013; B’Krong et al., 2018; Suresh et al., 2018
	E-20	M/AFP	Tapparel et al., 2013; Suresh et al., 2018
	E-21	E/M/AFP	Holmes et al., 2016; Suresh et al., 2018; Ramalho et al., 2019; Sun et al., 2019
	E-22	AFP	Suresh et al., 2018
	E-24	E/M/AFP	Tapparel et al., 2013; B’Krong et al., 2018; Suresh et al., 2018
	E-25	E/M/AFP	Holmes et al., 2016; B’Krong et al., 2018; Suresh et al., 2018; Ramalho et al., 2019; Sun et al., 2019
	E-26	AFP	Suresh et al., 2018
	E-27	E/M/AFP	Tapparel et al., 2013; B’Krong et al., 2018; Suresh et al., 2018
	E-29	M/AFP	Tapparel et al., 2013; Suresh et al., 2018
	E-30	E/M/AFP	Holmes et al., 2016; Mao et al., 2016; Chen et al., 2017; B’Krong et al., 2018; Suresh et al., 2018; Ramalho et al., 2019; Sun et al., 2019
	E-31	M/AFP	Tapparel et al., 2013; Suresh et al., 2018
	E-32	M/AFP	Tapparel et al., 2013; Suresh et al., 2018
	E-33	E/M/AFP	Tapparel et al., 2013; Suresh et al., 2018; Sun et al., 2019
	EV-B73	AFP	Suresh et al., 2018
	EV-B74	AFP	Suresh et al., 2018
	EV-B75	AFP	Tapparel et al., 2013; Suresh et al., 2018
	EV-B77	AFP	Tapparel et al., 2013; Suresh et al., 2018
	EV-B79	AFP	Suresh et al., 2018
	EV-B80	AFP	Suresh et al., 2018
	EV-B81	AFP	Tapparel et al., 2013; Suresh et al., 2018
	EV-B85	AFP	Tapparel et al., 2013; Suresh et al., 2018
	EV-B86	AFP	Tapparel et al., 2013; Suresh et al., 2018
	EV-B87	AFP	Tapparel et al., 2013; Suresh et al., 2018
	EV-B88	AFP	Tapparel et al., 2013; Suresh et al., 2018
	EV-B93	AFP	Tapparel et al., 2013; Suresh et al., 2018
	EV-B97	AFP	Tapparel et al., 2013; Suresh et al., 2018
	EV-B100	AFP	Tapparel et al., 2013; Suresh et al., 2018
	EV-B106	AFP	Suresh et al., 2018
	EV-B107	AFP	Suresh et al., 2018
C	CV-A1	AFP	Tapparel et al., 2013
	CV-A11	E/M/AFP	Tapparel et al., 2013; Suresh et al., 2018
	CV-A13	E/M/AFP	Tapparel et al., 2013; Suresh et al., 2018
	CV-A17	M/AFP	Tapparel et al., 2013; Suresh et al., 2018
	CV-A20	AFP	Tapparel et al., 2013; Suresh et al., 2018
	CV-A21	AFP	Tapparel et al., 2013
	CV-A22	M/AFP	Suresh et al., 2018
	CV-A24	M/AFP	Tapparel et al., 2013; Suresh et al., 2018
	EV-C96	AFP	Tapparel et al., 2013; B’Krong et al., 2018; Suresh et al., 2018
	EV-C99	AFP	Suresh et al., 2018
	EV-C109	AFP	Tapparel et al., 2013
D	EV-D68	E/M/AFP	Tapparel et al., 2013; Messacar et al., 2018; Suresh et al., 2018
	EV-D70	E/M/AFP	Tapparel et al., 2013; Suresh et al., 2018
	EV-D94	AFP	Tapparel et al., 2013; Suresh et al., 2018

*E, encephalitis; M, meningitis; AFP, acute flaccid paralysis.*

## Enterovirus Life Cycle

The enterovirus genome contains approximately 7.5 kb with a single open reading frame flanked by 5′- and 3′-untranslated regions (UTR) and is enclosed in a capsid. While the 5′ end is covalently attached to a viral protein genome-linked (VPg) required for replication, the polyadenylated 3′ terminus is important not only for the negative-stranded RNA synthesis but also for translation and RNA stability (Zoll et al., 2009; Kempf and Barton, 2015). The life cycle of enteroviruses begins with binding to one or more specific receptors on the cell surface. The cell receptors for enteroviruses are quite divergent. For example, the cell surface receptor for PV is cluster of differentiation 155 (CD155), whereas EV-A71 has nine cell surface receptors including human scavenger receptor class B member 2 (hSCARB2), human P-selectin glycoprotein ligand 1 (hPSGL1), annexin II (Anx2), heparan sulfate, sialylated glycan, dendritic cell-specific ICAM3-grabbing non-integrin, vimentin, nucleolin and human tryptophanyl-tRNA synthetase (hWARS) (Baggen et al., 2018; Yeung et al., 2018). Receptor binding allows viruses to enter host cells via receptor-mediated endocytosis. There are several endocytic pathways that can mediate the entry of receptor-bound enterovirus particles at the plasma membrane, including macropinocytosis, clathrin-dependent endocytosis and clathrin-independent uptake such as caveolae-dependent and non-caveolae-dependent endocytosis (Marjomaki et al., 2015). The specific endocytic pathways that virus exploits to enter the cells depend on the viral species and serotype, host cell type and local microenvironment (such as pH and temperature). For instance, EV-A71 enters rhabdomyosarcoma (RD) cells through the clathrin-dependent pathway, whereas the caveolae-mediated pathway is used to enter Jurkat cells. EV-A71 exhibits distinct endocytic pathways in different host cells, partly because of the diversity of the EV-A71 surface receptors (Yamayoshi et al., 2014). Upon entry into host cells, virus uncoating releases its viral RNA genome into the cytoplasm and translation is initiated from an internal ribosome sequence in the 5′ UTR, which is called internal ribosome entry site (IRES). An IRES is a *cis-*acting RNA element that forms secondary and tertiary structures to allow cap-independent initiation of translation (Shih et al., 2011; Lee et al., 2017). Translation of viral RNA yields a single polyprotein, which is proteolytically processed by viral proteases to produce four capsid proteins (VP4, VP2, VP3, and VP1) required for virion packaging, and seven non-structural proteins (2A-2B-2C and 3A-3B-3C-3D^pol^) as well as some stable precursors, which function in virus replication and disrupting the host cellular immune system. Viral RNA replication is catalyzed by the virally encoded RNA-dependent RNA polymerases, 3D^pol^, which utilizes Vpg as a protein primer to initiate the replication process. The synthesis of viral RNA takes place on the virus-induced remodeling of intracellular membranes that form replication organelles and leads to double-stranded RNA formation, which in turn will be transcribed into positive single-stranded RNA (Hsu et al., 2010; van der Schaar et al., 2016). Newly synthesized RNA may either serve as a template for translation and replication or be packaged into new infectious virions.

## CNS Invasion

**FIGURE 1:**
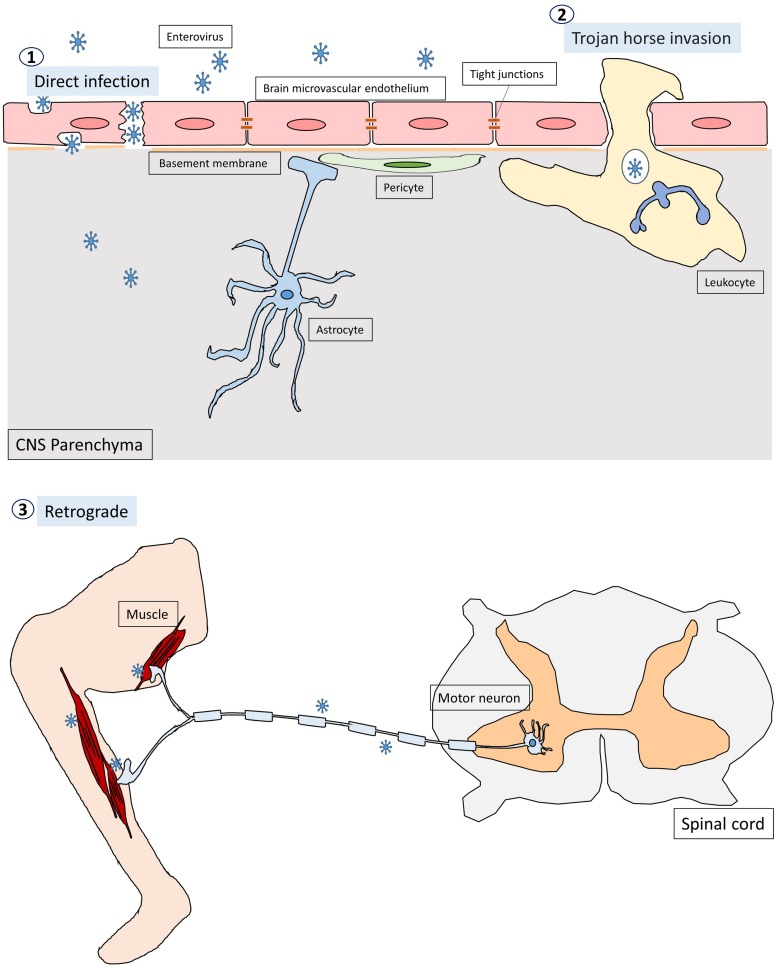
The pathways through which enteroviruses can gain access to the CNS. Enteroviruses can invade the CNS through three pathways. First, they infect BMECs, compromising and crossing the BBB to reach the CNS directly. The second pathway is known as Trojan horse route, in which enterovirus-infected leukocytes act as carriers and transverse the brain barriers bringing virus into the CNS. Thirdly, enteroviruses are able to hijack the retrograde axonal transport to enter the CNS from muscles into the motor neurons in the spinal cord.

Enteroviruses infect humans primarily through the fecal-oral route and replicate in the gastrointestinal tract with the exception of some enteroviruses, e.g., rhinovirus and EV-D68, which can cause respiratory infection and spread via respiratory secretion. After initial infection in the first exposed area, the enteroviruses can gain access to the CNS through multiple pathways, which are not mutually exclusive (Figure 1; Rhoades et al., 2011; Huang and Shih, 2015). First, most neurotropic viruses including enteroviruses spread through the bloodstream to reach the CNS. The spread of virus particles from the blood to the CNS is normally restricted by the blood brain barrier (BBB), which is a highly selective semipermeable barrier between the brain’s blood vessels and the cells in the brain. However, BBB integrity can be compromised by direct infection of the brain microvascular endothelial cells (BMECs) that make up the BBB or by the cytokines that are produced locally in the CNS during viral infections. For example, PV has been shown to invade the CNS via BBB transmission (Yang et al., 1997). Recent evidence has further demonstrated that mouse transferrin receptor 1 is responsible for PV attachment to the cell surface of BMECs, allowing invasion into the CNS via the BBB (Mizutani et al., 2016). Secondly, enteroviruses can invade the CNS through the peripheral circulating immune cells, which carry intracellular viruses (Tabor-Godwin et al., 2010). This is known as the Trojan horse route. Although the brain has been considered a site of immune-privilege, it has an active immune surveillance system that involves the recruitment of non-specific leukocytes such as phagocytes and lymphocytes into the meninges and cerebrospinal fluid (CSF) (Forrester et al., 2018). Indeed, it has been shown that the CSF contains a trafficking population of mononuclear cells, consisting of T cells (∼90%), B cells (∼5%), monocytes (∼5%), and dendritic cells (<1%) (Ransohoff and Engelhardt, 2012). Once these leukocytes are infected, they can act as carriers to bring viruses into the CNS. For instance, CV-B3-infected myeloid cells have been shown to cross the blood-CSF barrier in the choroid plexus (Tabor-Godwin et al., 2010). Upon entry into the CNS, the virus is likely released from myeloid cells and subsequently infect neurons and/or glia in the brain. There is also evidence that EV-A71 can infect leukocytes through binding to hPSGL1, a sialomucin membrane protein primarily expressed on leukocytes (Nishimura et al., 2009). Whether EV-A71-infected leukocytes can bring viruses into the CNS is not clear. Thirdly, certain enteroviruses can enter the CNS through peripheral nerves via retrograde axonal transport and *trans-*synaptic propagation (Gromeier and Wimmer, 1998; Chen et al., 2007; Ong et al., 2008). Axonal transport is an essential cellular process in neurons required for the movement of synaptic vesicles, lipids, proteins, and organelles including mitochondria, lysosomes, autophagosomes, and endosomes, to and from the cell body. It is well known that some neurotropic viruses can hijack the retrograde axonal transport to invade the CNS. For example, studies have shown that intramuscularly inoculated PV is taken up by endocytosis at the neuromuscular junctions (Ohka et al., 2004). The endocytosed viral particles in the axon terminal are moved in the retrograde direction toward the cell body via dynein-mediated vesicular transport without initiating uncoating (Ohka et al., 2009). The uncoating event takes place upon arrival at the cell body of the motor neuron. EV-A71 and EV-D68 can also enter and infect the CNS by retrograde axonal transport via peripheral spinal motor nerves (Chen et al., 2007; Ong et al., 2008; Hixon et al., 2019). Interestingly, a recent report has demonstrated that EV-A71 can directly infect the brainstem via cranial nerves, suggesting that the virus can use not only the motor components of spinal nerves but also cranial nerves to enter the CNS (Tan et al., 2014).

## Tropism

**FIGURE 2:**
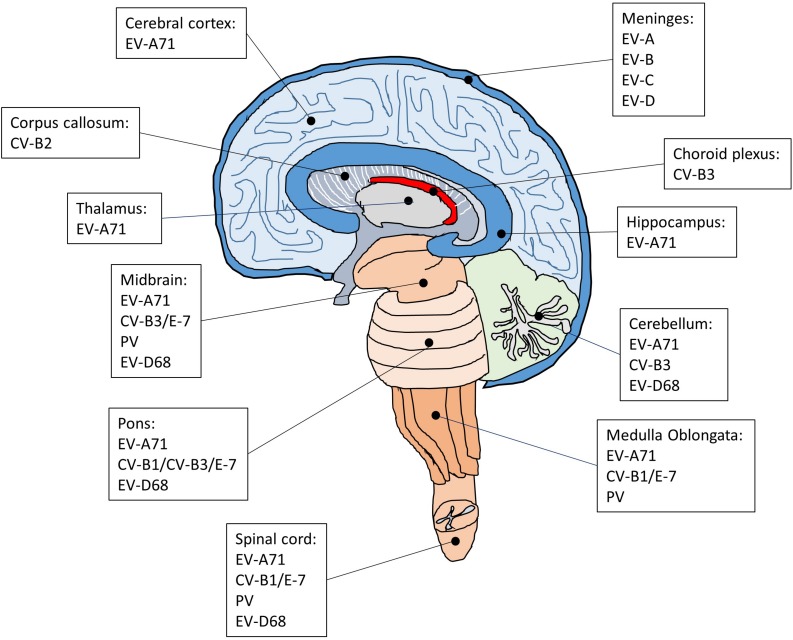
The regions of the CNS affected by enteroviruses that cause encephalitis, meningitis and AFP. The CNS is composed of cerebrum, diencephalon, cerebellum, brainstem, and spinal cord. Following enteroviral infection of the CNS, inflammation can occur in different anatomical regions such as the brain (encephalitis), meninges (meningitis), and spinal cord (myelitis), or simultaneously in multiple regions (meningoencephalitis, encephalomyelitis).

Each enterovirus has a distinct tropism that is determined by a combination of host and virus factors (Figure 2; Lin and Shih, 2014). Although neurotropic enteroviruses can invade the CNS and are associated with neurological disorders, dissemination of the virus to the CNS seems to occur sporadically and it is not clear how enterovirus targets specific regions and cell types in the brain and spinal cord. Earlier studies on poliovirus have suggested that virus tropism is determined by the cellular receptor for virus entry (Holland, 1961). However, the PV receptor CD155 is found in tissues that are not sites of PV infection (Mendelsohn et al., 1989; Freistadt et al., 1990; Koike et al., 1990), indicating that the cellular receptor is required for susceptibility to PV infection but not the sole determinant for virus tropism. Subsequent studies have suggested that the tissue-specific activity of IRES on viral RNAs also plays an important role in determining virus tropism (Gromeier et al., 1996; Yanagiya et al., 2003). For example, a chimeric PV carrying the IRES of hepatitis C virus replicates well in the liver but not in the brain of a mouse model for poliomyelitis, whereas the control poliovirus replicates well both in the liver and brain (Yanagiya et al., 2003). There is also evidence that innate immune antiviral activities such as the interferon (IFN) response is critical for virus tropism (Wessely et al., 2001; Ida-Hosonuma et al., 2005). In transgenic mice containing human CD155, PV replicates and produces severe lesions in the brain and spinal cord, whereas other tissues did not show severe pathological changes. However, in the CD155 transgenic mice lacking alpha/beta IFN, severe lesions are detected in the liver, spleen, and pancreas in addition to the CNS, suggesting that the alpha/beta IFN system is an important determinant for the differential susceptibility of tissue to PV. Poliovirus invades the CNS, which leads to the development of a paralytic disease in about 1% of virus-infected people (Melnick, 1996). Therefore, it has been suggested that, in 99% of the infected cases, the IFN response limits PV replication in extraneural tissues to prevent the invasion of the CNS (Racaniello, 2006). In the CNS, PV infects and replicates mainly in motor neurons in the anterior horn of the spinal cord, resulting in poliomyelitis (Nagata et al., 2004; Arita et al., 2006). In the most severe cases, PV attacks the neurons of the brainstem, causing bulbar poliomyelitis. In addition to neurons, the CNS contains three major types of non-neuronal cells called glial cells, including astrocytes, oligodendrocytes, and microglia. These glial cells play critical roles in maintaining homeostasis, myelin formation and providing support and protection for neurons. Interestingly, astrocytes and oligodendrocytes are also susceptible to PV infection in primary mouse culture prepared from the cerebral cortex of neonatal CD155 transgenic mice (Couderc et al., 2002). Whether these glial cells are targets for PV infection in the human brain is not known.

Although EV-A71 also invade the CNS, the infected areas are quite distinct compared with poliovirus. Brainstem encephalitis is the most common neurological presentation of EV-A71 infection. Consistently, viral lesions are mainly observed in the brainstem and predominantly located in the ventral, medial and caudal areas of the medulla oblongata (Kao et al., 2004). A few lesions are also found in the cortex, cerebellum, and spinal cord. In severe cases of EV-A71 infection, the major histopathological changes in the CNS are characterized by inflammatory damage, which selectively leads to neurogenic pulmonary edema and cardiac failure. In agreement with the main CNS lesion locations of EV-A71 infection, the neurons in the medulla oblongata have been implicated in the onset of neurogenic pulmonary edema (Davison et al., 2012). In addition, several autopsy results have suggested that EV-A71 may infect neurons and cause neuronal degeneration, which would activate inflammatory responses in the lesion area and cause encephalitis (Yan et al., 2000; Khong et al., 2012; Yao et al., 2012). Indeed, studies have shown that neurons are susceptible to EV-A71 infection (Huang et al., 2014; Feng et al., 2016). Intriguingly, although neurons can be infected by EV-A71, neural progenitor cells and astrocytes appear to be the main targets for EV-A71 infection in the CNS (Huang et al., 2014; Feng et al., 2016). Both of these cell types are similar in that they are capable of mitosis, which may be critical for virus replication (Yu et al., 2015). Neural progenitor cells are the progenitor cells of the CNS that give rise to many, if not all, of the neuronal and glial cell types and are important for many brain functions including learning and memory and cognition. Thus, the loss of neural progenitor cells due to EV-A71 infection may cause long-term abnormalities of the CNS. This is supported by a long-term follow-up study showing that a large proposition of children after EV-A71 infection with severe CNS involvement and cardiopulmonary failure exhibited delayed neurodevelopment and reduced cognitive function (Chang et al., 2007). Astrocytes also perform many functions in the brain, such as contribution to the formation of the blood-brain barrier, maintenance of extracellular ionic and chemical homeostasis and involvement in the injury response. Since astrocytes are mitotic and localized in a much broader area in the brain than neural progenitor cells, the preferential infection of astrocytes over neurons may create a reservoir for viral proliferation and enable the viral progeny to quickly spread in the CNS and induce massive inflammatory responses. It would be of interest to understand how EV-A71 show a preference for neural progenitor cells and astrocytes.

Similar to EV-A71, coxsackievirus has also been shown to infect neural progenitor cells in addition to neurons (Feuer et al., 2005). Studies have revealed that CV-B3 can infect proliferating neural progenitor cells located in the neonatal subventricular zone and hippocampus. Interestingly, CV-B3 preferentially replicates and induces cytopathic effects in undifferentiated neural progenitor cells (Tsueng et al., 2011). CV-B3 mediated loss of neural progenitor cells leads to a rapid decline in neurogenesis and may eventually cause developmental defects and CNS dysfunction (Ruller et al., 2012). However, the determinants of CV-B3 tropism in the CNS remain unclear. CV-B3 binds to target cells through two main receptors: decay-accelerating factor (DAF) (Bergelson et al., 1995) and coxsackievirus and adenovirus receptor (CAR) (Bergelson, 2009), which has been found to be highly expressed in the developing brain (Xu and Crowell, 1996). There is evidence that immature neurons express relatively high levels of CAR compare to their fully differentiated counterparts (Ahn et al., 2008), suggesting that the level of virus receptor is one of the critical determinants in preferential virus replication in undifferentiated neural progenitor cells. Besides, CAR exists as multiple isoforms and a specific isoform of CAR that is expressed at high levels in human pancreatic beta cells has been suggested to be prone to coxsackievirus infection (Ifie et al., 2018). Whether this specific isoform of CAR plays a role in coxsackievirus infection in the CNS is not known.

## Persistent Infection

Though enteroviruses have been considered as cytolytic viruses and diseases caused by infection with enteroviruses are typically short-lived, several studies have indicated that some enteroviruses may be associated with lifelong disorders including post-polio syndrome (Muir et al., 1995; Julien et al., 1999), schizophrenia (Rantakallio et al., 1997; Suvisaari et al., 2003; Khandaker et al., 2012), amyotrophic lateral sclerosis (Woodall et al., 1994; Berger et al., 2000; Giraud et al., 2001), type 1 diabetes (Richardson and Morgan, 2018), and chronic viral cardiomyopathy (Chapman and Kim, 2008). The cause of these enterovirus-associated lifelong disorders is still not clear, but it has been hypothesized that persistent infection of enterovirus may occur based on the presence of enterovirus RNA and protein in the affected tissues at stages of disease after acute infection (Chapman and Kim, 2008). Consistently, the spread of enteroviruses within tissues is not always accompanied by cell death (Bird et al., 2014). Two major groups of persistent infection have been observed *in vitro* (Pinkert et al., 2011). One group, called steady-state infection, is characterized by infection of all cells, while the other group, named carrier-state infection, is characterized by a cytolytic infection of a small proportion of cells, which spares the majority of cells in culture from cytolysis. There is evidence that enteroviruses establish carrier-state infection *in vitro* (Heim et al., 1992; Heim et al., 1995; Pinkert et al., 2011). The carrier-state infection is thought to be induced by the selection of virus mutants that are less cytopathic and may involve the coevolution of both cells and viruses. For example, following CV-B3 infection, expression of the CV-B3 receptor CAR has evolved to be downregulated or eliminated in a subpopulation of cells (Pinkert et al., 2011), which is known to be associated with a decrease of CV-B3 infection and cell lysis (Werk et al., 2005; Fechner et al., 2007). As a result, these evolved cells are protected from virus infection and cell lysis and become dominant in the culture within several passages. In the meantime, the virus also adapts to persist by gaining a CAR-independent entry mechanism (Pinkert et al., 2011). Thus, during the development of persistent infection, viruses and cells have co-evolved such that cellular resistance to viral replication is balanced. In addition to the coevolution model, other viral genomic alterations that cause persistent infection have been reported in the highly conserved 5′ UTR, which is critical for viral replication. For instance, CV-B mutants with deletions in the 5′ end of the viral genome persist in host tissues, and RNA of the variants can be stably detected from heart tissue of mice experimentally inoculated with wild type CV-B3 and from human cases of myocarditis (Kim et al., 2005; Chapman et al., 2008; Kim et al., 2008), suggesting that viral replication is important for persistent infection *in vivo*. Nonetheless, it has also been suggested that persistence of CV-B1 RNA in skeletal muscle or CV-B3 RNA in the CNS is not facilitated by genetic alterations that give rise to replication-defective forms, but occurs primarily through formation of stable and atypical double-stranded RNA complex (Tam and Messner, 1999; Feuer et al., 2009). Interestingly, besides the reduction of CAR, other cellular factors have also been shown to play an important role in persistent infection of CV-B3. In particular, CV-B3 replication is affected by the cell cycle status, suggesting that the persistence of CV-B3 may dependent on infection of quiescent cells in which viral replication is lowered or suppressed (Feuer et al., 2002; Feuer et al., 2004). Therefore, the differences between these mechanisms are likely due to distinct patterns of virus-host interactions and there may be tissue- or cell-specific mechanisms for establishing a persistent infection.

Early viral infection of the CNS can cause severe physical and intellectual disability and, in some cases, results in unexpected neurological disorders years after acute infection. For example, approximately 30 percent of polio victims experience new symptoms (post-polio syndrome) about 50 years after the primary infection. Some studies have shown that post-polio syndrome is correlated with the presence of viral RNA in the CNS, suggesting that PV can persist and cause long-term damage in the CNS (Muir et al., 1995). In consistent with these findings, it has been shown that poliovirus strains are not fully lytic in neuroblastoma cell lines (Colbere-Garapin et al., 1989). Following PV infection, massive cytopathic effects are observed in cultured cells, but some cells survive infection without further observable cytopathic effects in spite of continuous viral production. During persistent infection, PV mutants are constantly selected, and many of the identified mutations occur at positions known to be involved in the binding of PV receptors, suggesting that the interactions of the virus with its receptor is critical for the establishment of persistent infections (Colbere-Garapin et al., 1998). Although there is not a single mechanism for establishing a persistent infection, it is believed that the virus must evade host’s antiviral immune response. Because enteroviruses have high mutation rates due to the lack of proofreading ability in RNA polymerases, they can generate a variety of mutants not only to affect receptor binding and virus replication but also to evade the immune system. The CNS is relatively inaccessible to immune surveillance compared with other tissues, which makes it particularly vulnerable to persistent infection. Using *in vivo* imaging, a recent study has demonstrated that EV-A71 infection of AG129 mice, in which alpha/beta and gamma interferon receptors are deficient, shows rapid spread and long-term persistence of the virus in the brain of surviving animals (Caine and Osorio, 2017). Interestingly, high viral loads are maintained in the brain even at 6 weeks following infection, whereas viral loads in other tissues including heart, lung, liver, spleen and intestine are gradually reduced to a lower level. In agreement with these findings, *in vitro* studies have shown that in contrast to RD cells or neuroblastoma cell lines, motor neuron-like hybrid cell line (NSC-34) infected by EV-A71 does not display cytopathic effect and the viral particles adopt a non-lytic exit pathway through autophagy (Too et al., 2016). Furthermore, there is evidence that EV-A71 RNA is present in stool weeks after initial infection (Han et al., 2010). Taken together, all these findings suggest that similar to PV and CV-B3, EV-A71 can also establish a persistent infection in the CNS, but the long-term impact of EV-A71 infection in the CNS remains elusive.

## Neurovirulence

Some enteroviruses can cause diseases within the nervous system. The most thoroughly studied neurovirulent enterovirus is PV. To eradicate global poliomyelitis, much effort has been made to develop polio vaccines including an inactivated PV given by injection and an attenuated PV given by mouth. Although both types of polio vaccines are effective, oral polio vaccines are superior not only in administration but also in providing longer-lasting immunity. The attenuated PV was developed based on the observation that the virus no longer caused disease after many passages in different animals and cell cultures, but replicated sufficiently to induce protective immunity. Genetic analysis of the attenuated PV has shown that a point mutation within the IRES of the vaccine strains is a critical determinant of the attenuation phenotype (Evans et al., 1985; Kawamura et al., 1989; Ren et al., 1991). For instance, the C472U mutation in the IRES of poliovirus type 3 causes a translation defect, which leads to reduced replication in the CNS and attenuation of neurovirulence (La Monica and Racaniello, 1989; Gutierrez et al., 1997; Ohka and Nomoto, 2001). Subsequent studies have shown that the C472U mutation reduces the efficiency of binding of the polypyrimidine-tract binding protein (PTB) to the IRES, which is required for initiation of translation (Guest et al., 2004). Sequence analysis has also identified additional mutations in the capsid region of the vaccine strains. These capsid mutations are likely to disrupt viral particle binding to host cells and reduce the capsid stability, which may contribute to and stabilize the attenuation phenotype. However, PV is notably adaptable and attenuated viruses can increase its virulence through mutation and/or recombination (Jorba et al., 2008; Minor, 2009). Thus, although unusual, immunization with the attenuated vaccine strains may cause vaccine-associated paralytic poliomyelitis, which could be due to the reversion of the mutations in the viral genome that confer the attenuation phenotype and/or acquisition of new mutations with enhanced virulence (Kew et al., 2005; Famulare et al., 2016). Moreover, these vaccine-derived polioviruses (VDPV) have caused outbreaks of poliomyelitis in areas with low routine immunization rates (Burki, 2019). Using approach combining phylogenetic analysis of sequence data from outbreaks of VDPV and an experimental evolution approach in cell culture, a recent study has provided a model describing the evolutionary steps sufficient for the vaccine strain to lose its attenuation and become virulent (Stern et al., 2017). In the first step, the critical mutations for the attenuation phenotype are reverted to increase the viral replication. This is followed by recombination events with co-circulating enterovirus strains, most often a coxsackievirus strain, but in some cases a circulating PV strain to optimize viral replication in the human gut. In the final step, the virus continues to slowly revert to sequences that are conserved across wild-type PV to enhance the fitness of the virus. The information provides a powerful framework for developing safer vaccine strains and for forecasting virulence of viruses.

EV-A71 has emerged as a serious threat to public health across the Asia-Pacific region. EV-A71 causes hand, foot and mouth disease (HFMD) and herpangina, and occasionally severe neurological disorders. Unlike PV in which a point mutation within the IRES can attenuate neurovirulence, the association between EV-A71 neurovirulence and viral genome sequences remains largely unknown. So far, there are only a few reports showing that the neurovirulence can be weakened by mutations in the viral genome of EV-A71. For example, studies have shown that defined genetic manipulation of the EV-A71 genome based on the temperature-sensitive determinants of a poliovirus vaccine strain results in attenuated neurovirulence in monkeys (Arita et al., 2005). The generated EV-A71 mutant strain contains four mutations in the conserved regions of the enterovirus genome including one in the 5′ UTR, two in the 3D polymerase gene and one in the 3′ UTR, and all of them are required for substantial attenuation (Arita et al., 2008). Like PV, the capsid proteins also play an important role in EV-A71 virulence. For instance, the amino acid residue 145 of EV-A71 VP1, which affects receptor usage for cell surface attachment of the viral particles (Nishimura et al., 2013; Tan et al., 2017), has been shown to confer mouse adaptation with a G145E replacement (Arita et al., 2008; Chua et al., 2008) and influence the virulence in mice (Chua et al., 2008; Zaini and McMinn, 2012). Consistent with earlier results, a recent study has further demonstrated that the VP1-145 is a key determinant for EV-A71 neurovirulence in monkeys (Fujii et al., 2018). In addition, changes of nucleotides in the 5′ UTR and an amino acid replacement in the 2A or 3C protein have been identified to play an important role for virulence determination of EV-A71 (Li et al., 2011; Yeh et al., 2011; Li et al., 2017). Whether they attenuate EV-A71 neurovirulence is unknown. Nonetheless, it is expected that all these findings would contribute to not only our understanding of EV-A71 but also the development of live attenuated EV-A71 vaccine in the future.

EV-D68 is another non-polio enterovirus that has an association with a polio-like neurological disorder known as acute flaccid myelitis (AFM) with symptoms such as dysneuria and muscle weakness although the most common clinical symptom of EV-D68 infection is respiratory illness (Holm-Hansen et al., 2016; Messacar et al., 2018). EV-D68 is quite unique among enteroviruses because it carries some characteristic features of respiratory enteroviruses (Rhinovirus A-C) including optimal growth temperature of 33°C allowing better replication in the nasal cavity and inability to survive in the stomach due to acid sensitivity, but is genetically more closely related to enteric enteroviruses based on phylogenetic analysis. EV-D68 has become an emerging pathogen since an outbreak occurred in 2014 in the United States. Studies have shown that six mutations including M291T, V341A, T860N, D297N, S1108G, and R2005K are associated with neurovirulence of the outbreak EV-D68 strains causing AFM in 2014 (Greninger et al., 2015). Interestingly, another study has identified 3 nucleotide variables, C1817T, C3277A, and A4020G, in the 2014 outbreak strains, which differ significantly from previously identified EV-D68 strains (Huang W. et al., 2015). Among these three variables, C3277A causes amino acid substitution T860N in the protease 2A cleavage site between VP1 and 2A, whereas A4020G results in amino acid substitution S1108G in a protease 3C cleavage site between 2B and 2C, suggesting that mutation of these two sites may alter the cleavage efficiency and increase replication and transmission rates. However, using a mouse model of paralytic myelitis caused by EV-D68, a recent study has shown that the 2014 outbreak EV-D68 strains isolated from patients without AFM can also produce paralysis in neonatal mice (Hixon et al., 2017). Thus, further comparative analyses using infectious clones containing different combinations of previously identified mutations will be needed to establish the determinants of EV-D68 neurovirulence.

## Immune Responses in the CNS

About 3% of enterovirus infected people will develop encephalitis while most people never show evidence of CNS infection (Koskiniemi et al., 1991). Since encephalitis only occurs in a small percentage of infected people, it is thought that host-pathogen interactions and immune responses in peripheral sites prevent viruses from gaining access to and causing infection within the CNS. Patients with enterovirus encephalitis may present symptoms that range in severity from mild cognitive impairment and memory loss to permanent CNS damage and death. The symptoms are mainly caused by virus-induced inflammatory responses in the brain. Inflammation is the body’s protective immune response against infection, but the brain was thought to be immune-privileged based on two predominant beliefs. First, the BBB prevents the entry of circulating immune cells and antibodies into the brain. Secondly, there is no lymphatic drainage to alert the immune system to the presence of CNS antigens. However, these two beliefs have been questioned by the detection of small numbers of leukocytes in the CSF and the newly identified lymphatics that allow leukocyte egress (Engelhardt et al., 2017). In addition to the BBB, two other brain barrier structures have been described: (1) blood – CSF barrier to the choroid plexus, which is located at the ventricles of the brain, and (2) blood – CSF barrier to the pia arachnoid, which is located at the surface of the brain. It is important to note that CSF spaces in these two brain barriers do not exhibit the same immune privilege as the CNS parenchyma and contain diverse immune cells to monitor the CSF for the presence of immune signals in response to viral pathogens. Therefore, the immune privilege of the brain is not absolute but instead is relative to other organs.

Enterovirus encephalitis is reported to occur more frequently in younger children. Although the exact cause remains unknown, it has been suggested that a compromised or immature immune system may be involved in the observed effect. Studies have shown that expression of CD40-ligand on activated T cells and interleukin 4 (IL-4) production are significantly lower in EV-A71 infected children with meningoencephalitis than those without it (Yang et al., 2001). CD40-ligand plays a pivotal role in co-stimulation and regulation of the immune responses. It binds to the CD40 receptor on antigen-presenting cells including B cells and macrophages to facilitate cell-cell communication and modulate adaptive immunity. Interleukin-4 (IL-4) is also a key regulator in adaptive immunity. It is a cytokine that has many functions including the stimulation of activated B cell and T cell proliferation and the differentiation of naïve helper T cells and B cells into Th2 cells and plasma cells, respectively. Thus, a reduction in CD40-ligand and IL-4 may indicate that adaptive immunity is compromised. In addition, differences in the polymorphism of the cytotoxic T lymphocyte antigen-4 (CTLA-4) have been noted between children with and without meningoencephalitis in the same study (Yang et al., 2001). CTLA-4 is a surface receptor on T cells, which functions to downregulate T cell activity and polymorphism of CTLA-4 has been shown to be associated with certain autoimmune diseases (Marron et al., 1997). Interestingly, a recent study has reported a correlation between human leukocyte antigen (HLA) genotype and enterovirus infectivity in young children (Sioofy-Khojine et al., 2018). Consistently, there is evidence that genetic differences between individuals may affect the immune response to infection (Kim-Hellmuth et al., 2017). Taken together, these results suggest that genetic factors may confer susceptibility to neurological complications following enterovirus infection.

Although genetic susceptibility may provide clues to why enterovirus encephalitis only occurs in some children but not in others, it cannot explain why younger children infected with enteroviruses are at higher risk for developing encephalitis. It has been suggested that the immaturity of the BBB in early development may account, at least in part, for the age-related difference in viral neuroinvasion (Saunders et al., 2014). The development of the BBB is a multistep process that starts with the growth of new vessels into the embryonic neuroectoderm from pre-existing vessels (Blanchette and Daneman, 2015). This is followed by the expression of tight junction proteins and nutrient transporter in BMECs that lines blood vessels in the brain. The BBB becomes mature as nascent vessels come into close contact with pericytes and astrocytes, which provide structural and functional support to the BBB. In addition, the other cell types present at the BBB, including neurons, microglia, and perivascular macrophages also contribute to the properties of the BBB (Banerjee and Bhat, 2007). Interestingly, it has been shown that the BBB is capable of restricting entry of proteins and small molecules during embryogenesis prior to postnatal astrocyte generation and ensheathment of the vessels (Daneman et al., 2010; Saunders et al., 2014), suggesting that the BBB is functionally mature even without astrocytes in early development. Although astrocytes are not required for initial BBB formation, there is strong evidence that astrocytes play an important role in regulating the function of the BBB during postnatal development (Haseloff et al., 2005). For example, astrocytes secrete trophic factors that lead to tighter tight junctions between BMECs (Dehouck et al., 1990; Rubin et al., 1991). In addition to its role in regulating the BBB, astrocytes receive signals from neighboring neurons and responding to them with the release of neuroactive substances to modulate synaptic strength in the CNS (Santello et al., 2019). Moreover, there is also evidence that astrocytes convey signals from neurons to the vasculature, leading to arteriolar dilation and an increase in local blood flow (Anderson and Nedergaard, 2003; Zonta et al., 2003). Thus, astrocytes link neuronal activity to functional properties of the BBB. Given that astrocytes are critical for structural support and the maintenance of the BBB, the developing brain containing immature astrocytes may have higher BBB permeability compared to that in the adult brain and is likely to be more vulnerable to viral neuroinvasion.

The CNS immune system may also be involved in the age-related difference in developing enterovirus encephalitis. Microglia, the primary resident immune cells of the brain, play a key role in regulating signaling pathways during CNS inflammation (Rivest, 2009). Although microglia are phenotypically and developmentally different from peripheral macrophages, they use phagocytic and cytotoxic mechanisms to destroy foreign pathogens and act as antigen-presenting cells to initiate T cell-mediated adaptive immune responses similar to macrophages. In addition, microglia have been shown to induce the recruitment of monocytes into the brain during viral infection (Fekete et al., 2018). However, whether microglia are fully functional during early development is not clear. Microglia in the early postnatal brain exhibit different morphologies compared to the adult brain (Cuadros and Navascues, 1998). They are largely non-ramified and take on an ameboid shape during early development, and gradually differentiate into mature/ramified microglia as the brain mature. Consistently, distinct sets of genes are expressed in microglia during different phases of development (Bennett et al., 2016; Matcovitch-Natan et al., 2016). The ameboid morphology observed in developing microglia is similar to activated phagocytic microglia from the adult brain, suggesting that microglia are in a constitutively “activated” state in the developing brain (Lenz and Nelson, 2018). Interestingly, it has been shown that activated microglia produce inflammatory cytokines such as TNFα and IL-1β (Nishioku et al., 2010; Yang et al., 2015), that increase BBB permeability and downregulate tight-junction proteins between BMECs (Gu et al., 2015; Almutairi et al., 2016). Taken together, these results suggest that developing microglia may increase BBB permeability by releasing inflammatory cytokines. Intriguingly, another study has shown that gene expression profiles are distinct between activated microglia from the adult brain and microglia from a control neonatal brain (Wlodarczyk et al., 2017). Therefore, developing microglia are not the same as adult “activated” microglia even both of them exhibit a remarkable similarity in morphology. Nonetheless, this may provide a logical explanation of why younger children infected with enteroviruses are at higher risk for developing encephalitis.

## Conclusion

Enterovirus infection is a major public health concern considering the increase in outbreaks of serious neurological complications. Although there has been considerable progress in studying the complex interplay between enteroviruses and the infected cell in a culture dish, more complicated interaction between virus and host *in vivo* is largely unknown and how enteroviruses gain access to and spread in the well-protected CNS remains to be explored. For example, a common feature of enteroviral encephalitis is the involvement of brainstem (Wasserstrom et al., 1992; Huang et al., 1999; Shen et al., 1999; Lum et al., 2002; Brecht et al., 2010; Fan and Liu, 2019), but some studies have revealed cerebral white matter lesions without brainstem involvement in neonatal enteroviral encephalitis (Verboon-Maciolek et al., 2006; Hirata et al., 2011; Wu et al., 2014; Correia et al., 2016), suggesting that age and brain maturation may play an important role in the pathogenesis of enteroviral encephalitis. In the current review, we propose that there is a causal link between the development of the neuroimmune system and enteroviral neuroinvasion and have suggested that the age-related difference in developing enterovirus encephalitis may be associated with the development of neuroimmune system such as maturation of astrocytes and/or microglia. Future work will need to include the further characterization of the complex interactions between host and enteroviruses using appropriate animal models and the developmental roles of astrocytes and microglia in regulating BBB permeability. We also need to better understand the regulation of immune responses in the CNS caused by enteroviruses.

## Author Contributions

All authors contributed to the writing and discussion of this review article and approved the final version of the manuscript. H-CL made the figure and table.

## Conflict of Interest

The authors declare that the research was conducted in the absence of any commercial or financial relationships that could be construed as a potential conflict of interest.
